# *S*-Adenosylmethionine affects ERK1/2 and STAT3 pathway in androgen-independent prostate cancer cells

**DOI:** 10.1007/s11033-022-07331-2

**Published:** 2022-03-18

**Authors:** Thomas Schmidt

**Affiliations:** 1 Institute of Anatomy and Clinical Morphology, University of Witten/Herdecke, 58448 Witten, Germany; 2 Present Address: Department of Anatomy and Developmental Biology, CBTM, Medical Faculty Mannheim, Heidelberg University, Ludolf-Krehl-Strasse 7-11, Mannheim, Germany

**Keywords:** Androgen independent, Prostate cancer, *S*-Adenosylmethionine, Erk1/2, STAT3

## Abstract

**Background:**

The most critical point in the treatment of prostate cancer is the progression towards a hormone-refractory tumour, making research on alternative therapies necessary. This study focused on the methyl donor *S*-adenosylmethionine (SAM), which is known to act as an antitumourigenic in several cancer cell lines. Though a genome-wide downregulation of proto-oncogenes in prostate cancer cell lines treated with SAM is obvious, the anticancer effects remain elusive. Thus, in this study, the impact of SAM treatment on the cell cycle, apoptosis and cancer-related pathways was investigated.

**Methods and results:**

After performing SAM treatment on prostate cancer cell lines (PC-3 and DU145), a cell-cycle arrest during the S-phase, a downregulation of cyclin A protein levels and an upregulation of p21 cell cycle inhibitor were observed. The proapoptotic Bax/Bcl-2 ratio and the caspase-3 activity were elevated; additionally, the apoptosis rate of SAM treated cells increased significantly in a time-dependent manner. Moreover, immunoblots displayed a downregulation of Erk1/2 and STAT3 phosphorylation accompanied by a reduced expression of the STAT3 protein.

**Conclusion:**

SAM caused changes in cancer-related pathways, probably leading to the effects on the cell cycle and apoptosis rate. These results provide deeper insights into the anticancer effects of SAM on prostate cancer cells.

## Introduction

Prostate cancer is the second most common type of malignancy in males. One in nine males will be diagnosed with prostate cancer during their lifetime [1]. Treatment options comprise surgery, radiation therapy and chemotherapy as well as androgen ablation, all of which may be accompanied by a reduction in quality of life for the patients [2]. Moreover, many patients relapse within 2 years, undergoing a progression towards a hormone-refractory tumor [2]. Thus, extensive research focused on alternative therapies has been initiated over the last decades.

*S*-Adenosylmethionine (SAM), also known as Ado-Met, represents the most important biological methyl donor [3]. Its synthesis is catalysed by methyl-adenosine transferase (MAT) in the cytosol and presumably also in the nucleus of most living cells [4]. Two genes encoding for MAT have been described so far, *MAT1A* which is expressed exclusively in the liver and encodes for the alpha1 subunit as well as *MAT2A*, which is not cell-type-specific and encodes for the alpha2 subunit [4, 5]. The variable combination of these subunits forms three isoforms of methyl-adenosine transferases differentially distributed in the body tissues [4, 5]. Generally, SAM is involved in three essential metabolic pathways, i.e. transsulfuration, polyamine biosynthesis and especially transmethylation [6, 7]. In humans, over 50% of the methionine intake is transformed to SAM, mostly in the liver, where 85% of the methylation reactions take place [8]. Not surprisingly, SAM is involved in the transfer of methyl groups to a broad variety of nucleophils, comprising oxygen, nitrogen, carbon atoms on proteins, lipids, carbohydrates and last but not least nucleic acids [7]. The transfer of methyl groups from SAM leads to the demethylation of the molecule and to the formation of *S*-adenosylhomocysteine (SAH or AdoHcy) which acts as an inhibitor of methylation reactions [3]. Thus, the ratio of SAM/SAH represents a metabolic switch, controlling the extent of intracellular methylation, e.g. DNA methylation. To maintain methylation reactions, AdoHcy has to be continually removed [9]. In animal models, an accumulation of Adohcy causes global demethylation of DNA [3], which is often found in the genomes of cancer cells. Further pathways link SAM to oncogenesis. Glutathione synthesis [3, 10], spermidine and spermine synthesis as well as the homeostasis of AdoMet, decarboxylated AdoMet, (dcAdomet), AdoHcy, 5′-methylthioadenosine (MTA) and methionine seem to be critical in the development and the sustainability of cancer cells and preneoplastic lesions [3, 11–14]. To keep up this homeostasis, it is essential to control the intracellular concentration of SAM which in turn depends on methionine intake and the type of methyl-adenosine transferase (MAT) expressed in the cell [3, 14].

Apart from its function as a key factor in the mentioned metabolic reactions, SAM plays a pivotal role as a regulator of several essential cellular processes, e.g. proliferation, cell differentiation and apoptosis in vivo and in vitro [15–17]; however, in various cell lines, those processes are partially driven by totally different mechanisms. The treatment of colon cancer cells with SAM, for example, leads to the activation of procaspase-8, which is involved in apoptosis, by downregulation of cFLIP, a cellular FLICE inhibitory protein [18]. Recently, it has been reported, that the treatment of cancer cell lines with SAM leads to an increase of promoter remethylation of protooncogenes in several cancer cell lines accompanied by downregulation of those genes, leading to diminished proliferation, migration and invasion potential of treated cancer cells. Genome-wide modifications in methylation profiles of prostate cancer and colon cancer cell lines could not be detected; nevertheless, it is obvious that the treatment of cancer cells with SAM alters the transcription profiles of numerous proto-oncogenes [19, 20]. The exact mechanisms for these alterations are still not known and may be multifarious in different cancer entities. Due to this lack of knowledge, this study further investigates the underlying mechanisms of SAM actions in prostate cancer cells.

## Materials and methods

### Cell culture

Human prostate cancer cell lines PC-3 and DU145 were purchased from DSMZ-German Collection of Microorganisms and Cell Cultures GmbH, Braunschweig. The cells were seeded in 25 cm^2^ culture flasks in RPMI 1640 medium supplemented with 10% fetal calf serum (FCS) and 1.2% penicillin/streptomycin as described before [19] (PAN-Biotech GmbH, Aidenbach, Germany). Subsequently, they were treated either with vehicle (0.005 M H_2_SO_4_) or 200 µm of *S*-adenosylmethionine (SAM) (New England Biolabs, Ipswich, MA). The most effective concentration of SAM for both cell lines was determined in a previous study [19]. Treatment with vehicle or SAM was carried out by direct addition to the regular growth medium under sterile conditions. The medium was changed every other day.

### Proliferation assay

The CellTiter 96® AQ_ueous_ Non-radioactive Cell Proliferation Assay (Promega, Mannheim, Germany) was used, according to the manufacturer’s instructions, to assess the proliferation of PC-3 and DU145 cells exposed to SAM. Briefly, PC-3 and DU145 cells were seeded in 96-well plates (2000 or 1500 cells per well respectively) and grown for 24 h. Subsequently, they were treated with 200 µm of SAM or the vehicle for 24 h, 72 h and 120 h, respectively. The medium was changed every other day. Proliferation was assessed by measuring the bioreaction of the tetrazolium MTT 3-(4,5-dimethylthiazol-2-yl)-2,5-diphenyltetrazolium bromide to a coloured formazan dye using a plate reader. At least three independent experiments were performed in triplicate. Data are expressed as percentages of proliferation, where the proliferation rates for the control were arbitrarily set to 100%.

### Western-blot analysis

Total protein samples from cultivated cells were created using a modified RIPA buffer recipe (2). Proteins were separated by SDS-Page, transferred to a polyvinylidene fluoride membrane and incubated with primary antibodies overnight at 4 °C. Primary antibodies are listed in Table 1. The secondary antibody was goat anti-rabbit IgG conjugated with horseradish peroxidase (Cell signalling technology). Chemiluminescence was determined using the ECL detection system (Pierce, Rockford, IL). Membranes were stripped and re-probed with antibodies against tubulin as a loading control. Western blot signals were detected by FluorChem Q (Biozym Scientific GmbH, Hessisch Oldendorf, Germany) and densitometrically analyzed using the associated software. All blots were performed in three independent experiments.

**Table 1:** Overview of antibodies used in this study

Antibody	Host animal	Source	Dilution
Phospho-p44/42 MAPK	Rabbit	Cell Signalling (9101)	1:1000
P44/42 MAPK	Rabbit	Cell Signalling (9102)	1:1000
STAT3	Rabbit	Cell Signalling (4904)	1:1500
Phospho-STAT3	Rabbit	Cell Signalling (9131)	1:1000
p21	Rabbit	Cell Signalling (2947)	1:500
Cyclin A	Rabbit	ThermoFisher (PA5-36048)	1:500
Bax	Rabbit	Cell Signalling (2772)	1:1000
Bcl-2	Rabbit	Cell Signalling (4223)	1:500
Tubulin	Rabbit	Cell Signalling (2144)	1:5000

### Fluorogenic caspase activity assay

PC-3 and DU145 cells were washed with PBS. Cell lysis buffer was added (#70108, Cell Signaling Technology, Danvers, MA, USA) and cell lysates were collected in Eppendorf cups. Subsequently, the caspase activity assay (Caspase-3 Activity Assay Kit #5723, Cell Signaling Technology, Danvers, MA, USA) was performed according to the manufacturer’s instructions. Fluorescence was measured using a plate reader (excitation wavelength: 380 nm; emission wavelength: 460 nm) and expressed in relative fluorescence units (RFU).

### Apoptosis and cell cycle analysis

Apoptosis was assessed using the Muse™ Annexin V and Dead Cell kit (Millipore, Billerica, MA, USA) according to the manufacturer’s instructions. Briefly, the PC-3 and DU145 cells were trypsinised and washed with phosphate-buffered saline (PBS). Subsequently, 100 µl of Muse Annexin V & Dead Cell reagent was added to a 100-μl cell suspension. After incubation in the dark for 20 min at room temperature, the samples were analysed by Muse™ Cell Analyzer (Merck, Millipore). All experiments were carried out in triplicate. The cell cycle distribution analysis was measured using a Muse Cell Cycle Assay Kit (Merck, Millipore) according to the manufacturer’s instructions. PC-3 and DU145 cells were harvested and washed with PBS. Subsequently, 200 µl of the suspended cells were fixed in 70% ethanol for 4 h at − 20 °C. Then, cells were centrifuged at 300×*g*, washed with PBS, dissolved in 200 μl of Muse cell cycle reagent, incubated for 30 min in the dark at room temperature and analysed by Muse™ Cell Analyzer (Merck, Millipore).

### Statistical analysis

All experiments were carried out at least in triplicate. Statistical analysis was performed using GraphPad Prism (GraphPad Software, Inc., CA, US). All data are expressed as mean with ± SD (Standard deviation). Results were compared using the student’s *t*-test. In general, P-values < 0.05 were considered significant.

## Results

### Treatment with SAM decreases the proliferation of prostate cancer cells

**Fig. 1:**
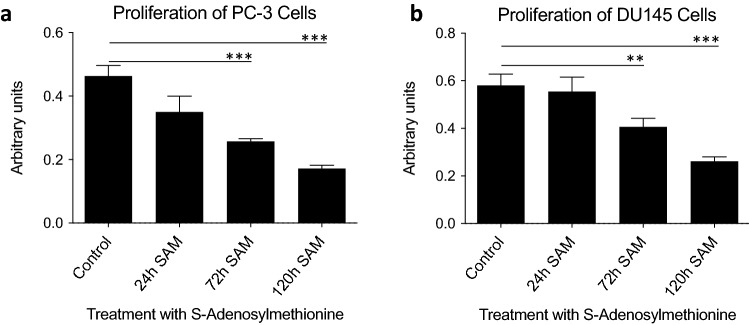
Treatment of prostate cancer cells with 200 µm *S*-adenosylmethionine (SAM) inhibits the proliferation in a time-dependent manner. PC-3 (**A**) and DU145 (**B**) cells were seeded in 96 well plates and treated with SAM or the vehicle (control) for 24 h, 72 h and 120 h respectively. Proliferation was measured using the 3-(4,5-dimethylthiazole-2-yl)-2,5-diphenyl tetrazolium bromide assay. Treatment of PC-3 and DU145 cells with SAM displayed an impaired proliferation compared to the control. The intensities of signals are expressed as arbitrary units. The image shows the means ± SD of three independent experiments: *P < 0.01, **P < 0.001, ***P < 0.0001

First, a proliferation assay was performed, to show that SAM inhibits the growth of prostate cancer cell lines, as previously described [19]. Time-course experiments displayed a diminished proliferation of PC-3 (Fig. 1A) and DU145 (Fig. 1B) cells treated with a concentration of 200 µm of SAM. After 24 h treatment, the proliferation decreased slightly compared to cells exposed to the vehicle (PC-3: 87 ± 4%; DU145 0.95 ± 5% of control). After 72 h and 120 h, SAM caused a significant reduction of proliferation of PC-3 and DU145 cells (PC-3: 48 ± 3% and 39 ± 4% of controls; DU145: 69.9 ± 5% and 45 ± 3%) (Fig. 1A, B).

### SAM causes cell cycle arrest in the S-phase of prostate cancer cells

**Fig. 2:**
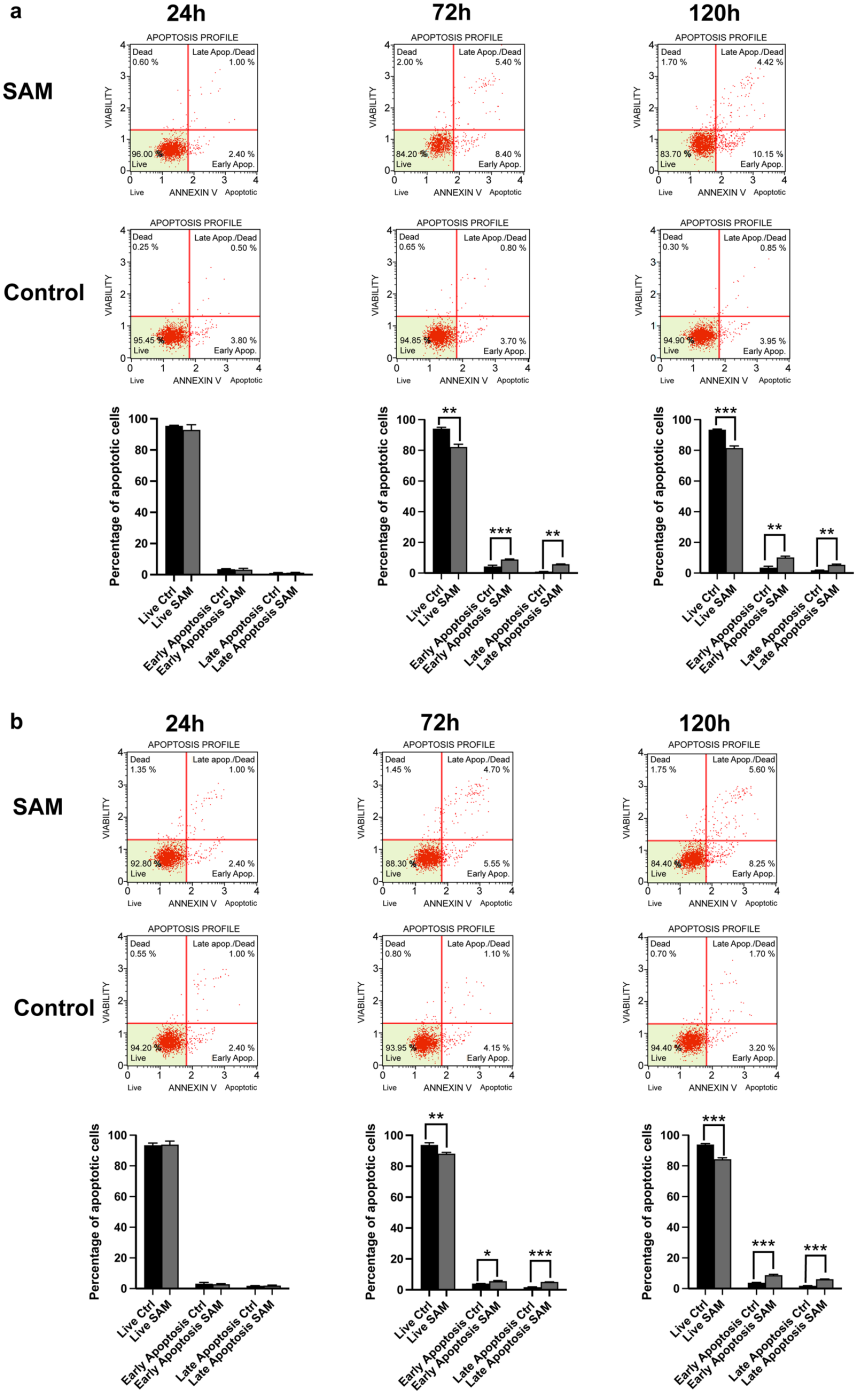
Cell cycle analysis of PC-3 (**A**) and DU145 (**B**) cells treated with 200 µm of *S*-adenosylmethionine. The treated cells were analysed after 24 h, 72 h and 120 h respectively using Muse™ cell analyser. **A** and **B** are showing representative cell cycle plots at different time points treated with SAM and the vehicle (Ctrl, vehicle). The quantitative data indicate a significant arrest of SAM treated prostate cancer cells in the S-phase after 72 h and 120 h compared to controls and a significant decrease of treated cells in the G0/G1 phase. Treated DU145 cells additionally display a significant accumulation during the G2/M phase. The images display the means ± SD of three independent experiments: *P < 0.01, **P < 0.001, ***P < 0.0001

To gain more information about the origin of the inhibitory effect, cell cycle analysis of treated and control samples was carried out using the Muse™ Cell Analyzer. Figure 2 displays the time-dependent distribution of PC-3 (Fig. 2A) and DU145 (Fig. 2B) cells in the different cell cycle phases following treatment with SAM (200 µm). After 24 h, no significant differences between treated cells and controls could be observed in both cell lines. After 72 h, a highly significant increase of cells in the S-phase (PC-3: 39 ± 5%; DU145: 31 ± 2%) compared to controls (PC-3: 25 ± 3%; DU145: 22 ± 2%) was found, accompanied by a significant decrease of treated cells in the G1/G0 phase (PC-3: 31 ± 4%; DU145: 50 ± 3%) compared to controls (PC-3: 50 ± 3%; DU145 69 ± 4%). After 120 h of treatment with SAM, there was even a higher percentage of cells accumulated in the S-phase (59 ± 6% of the PC-3 cells and 39 ± 3% of the DU145 cells) compared to controls (PC-3: 22 ± 2%; DU145: 20 ± 3). Additionally, the percentage of treated cells in the G0/G1 phase (PC-3: 25 ± 3%; DU145: 37 ± 3%) decreased in both cell lines compared to controls (PC-3: 60 ± 4%; DU145: 64 ± 5%). Both results were highly significant. Moreover, after 120 h of treatment with SAM, DU145 cells showed a highly significant accumulation in the G2/M phase (16 ± 2%) compared to controls (9 ± 1%).

### SAM induces apoptosis in prostate cancer cells in a time-dependent manner

**Fig. 3:**
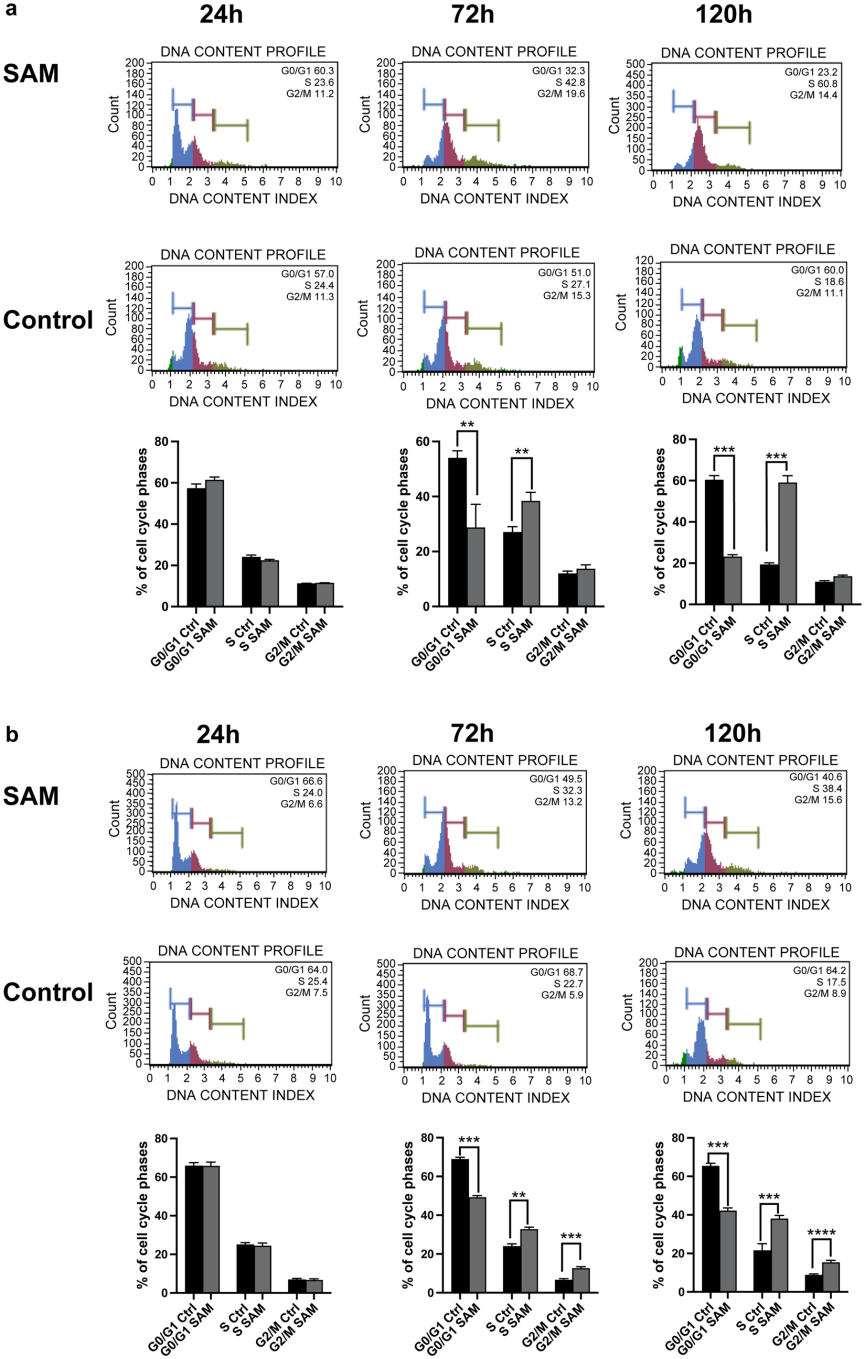
Distribution of the apoptotic cells was estimated by Muse™ cell analyser. PC-3 (**A**) and DU145 (**B**) cells were treated with 200 µm *S*-adenosylmethionine for 24 h, 72 h and 120 h. The percentage of early and late apoptotic cells was assessed and compared to controls (Ctrl). The quantification of the data shows a significant increase of early and late apoptotic cells and a decrease of living cells after 72 h and 120 h of treatment in both cell lines. The images display the means ± SD of three independent experiments: *P < 0.01, **P < 0.001, ***P < 0.0001

Figure 3 shows significant differences in the induction of apoptosis in PC-3 (Fig. 3A) and DU145 (Fig. 3B) cells following treatment with SAM compared to controls exposed to the vehicle. After 24 h, however, no alterations between controls and treated cells could be observed in both cell lines (Fig. 3A, B). In PC-3 cells treated with SAM, however, the percentage of living cells decreased significantly after 72 h (from 96 ± 2% to 83 ± 3%) and after 120 h (95 ± 2% to 82 ± 3%) A significant increase in the fraction of early apoptosis could be detected after 72 h and 120 h of treatment (SAM: 9 ± 1%/11 ± 2% compared to controls: 3.7 ± 0.6%/4 ± 0.9%) and also in the fraction of the late apoptosis (SAM: 6 ± 0.9%/5 ± 1% compared to controls: 1 ± 0.3%/1 ± 0.2%). In DU145 cells, the percentage of living cells decreased significantly after 72 h (Controls: 94 ± 6%; SAM: 88 ± 4%) and 120 h (Controls: 94 ± 4%; SAM: 84 ± 5%) of treatment with SAM. The fraction of early apoptosis in treated DU145 cells increased slightly after 72 h (controls: 4 ± 1%; SAM: 6 ± 1%) and more clearly after 120 h (controls: 3 ± 1%; SAM: 9 ± 1%), wheras the fraction of late apoptosis increased highly significantly after 72 h and 120 h, respectively (controls: 1 ± 0.2%/2 ± 0.4%; SAM: 5 ± 1%/6 ± 1%).

### SAM affects the expression of relevant cell cycle and apoptosis regulating proteins

**Fig. 4:**
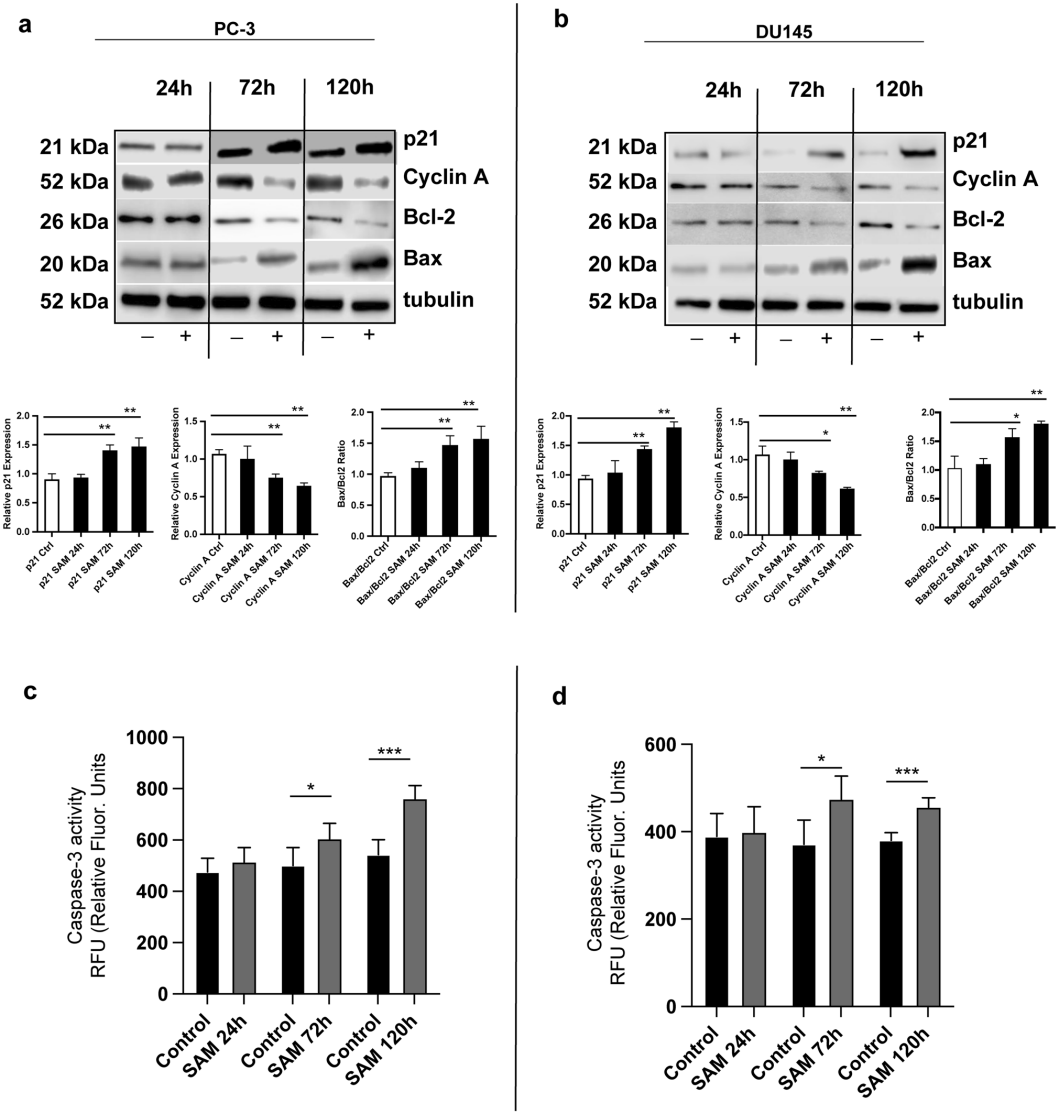
Differential expression of some essential cell cycle and apoptosis regulating proteins in PC-3 (**A**) and DU145 (**B**) cells treated with SAM (200 µm) or the corresponding controls (treated with the vehicle) for 24 h, 72 h and 120 h respectively. The Western blot analysis was carried out with antibodies against p21, Cyclin A, Bax and Bcl-2 Tubulin was used for the normalisation of target protein expression. The densitometric intensity of controls was compared to preparations of cells treated with SAM for the above-mentioned durations. The quantitative data display a significant increase of the Bax/Bcl-2 ratio and an increased expression of p21 protein in treated cells, whereas the expression of cyclin A protein decreased significantly in both cell lines. The intensities of signals are expressed as arbitrary units. A fluorogenic caspase activity assay revealed a significant upregulation of caspase-3 activity in PC-3 (**C**) and DU145 (**D**) cells after SAM treatment for 72 h and 120 h respectively. The images display the means ± SD of three independent experiments: *P < 0.01, **P < 0.001, ***P < 0.0001, ****P < 0.00001

These results were corroborated by the investigation of a choice of proteins relevant for the regulation of the cell cycle and apoptosis in treated PC-3 (Fig. 4A) and DU145 (Fig. 4B) cells and control samples. The expression of proapoptotic mitochondrial protein Bax increased, whereas the level of antiapoptotic protein Bcl-2 decreased after 72 h and 120 h of treatment. The Bax/Bcl-2 ratio increased significantly after 72 h and 120 h in both cell lines. Additionally, a significantly higher expression of *p21* in PC-3 and Du145 cells could be detected after 72 h and 120 h of treatment, whereas cyclin A was significantly downregulated.

### SAM treatment increases caspase-3 activity in PC-3 and DU145 cells

Next, a fluorogenic caspase-3 activity assay was peformed, wherein SAM treatment of PC-3 (Fig. 4C) and DU145 (Fig. 4D) cells significantly induced caspase-3 activity after 72 h and 120 h, respectively; however, a significant increase after 24 h of treatment could not be detected.

### SAM treatment affects the expression and phosphorylation of STAT3 and MAPK (ERK1/2) proteins

**Fig. 5:**
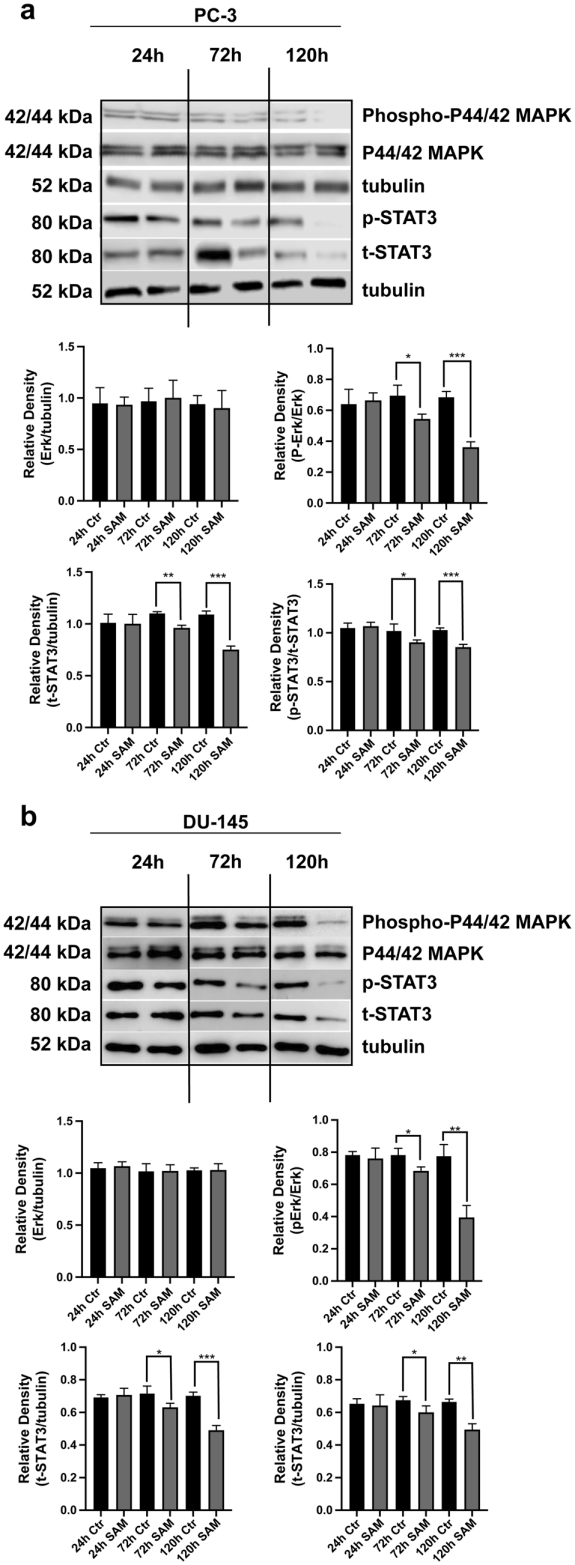
Effects of SAM treatment on the expression and phosphorylation (activation) of ERK1/2 and STAT3 proteins assessed by SDS PAGE. Western blots using cell preparations of PC-3 (**A**) and DU145 (**B**) cells treated with SAM (200 µm) for 24 h, 72 h and 120 h respectively and controls (vehicle) were carried out with the appropriate antibodies. The analysis of the densitometric intensity of the treated samples compared to untreated controls showed a significant decrease of phosphorylated p42/44 after 72 h and 120 h treatment with SAM in both cell lines; moreover, STAT3 and phosphorylated STAT3 protein were significantly decreased after 72 h and 120 h of treatment with SAM in PC-3 and DU145 cells. Intensities of signals are expressed as arbitrary units. The images display the means ± SD of three independent experiments: *P < 0.01, **P < 0.001, ***P < 0.0001, ****P < 0.00001

For further investigations of SAM effects on prostate cancer cells, Western-blot studies on two essential pathways relevant for the development of cancer were carried out. The analysis of the extracellular signal-regulated kinases, ERK1/2, showed a significant reduction of the phosphorylation which means a reduced activation of the pathway after 72 h and 120 h of treatment with SAM in both cell lines (Fig. 5A, B). Looking at the STAT3 pathway, a significant reduction of the total STAT3 protein could be observed after 72 h and 120 h of treatment in both cell lines. The phosphorylation of the STAT3 protein, however, was significantly decreased after 72 h and 120 h of SAM treatment.

## Discussion

Due to the lack of effective therapy options concerning androgen receptor-negative prostate cancer and bone metastasis, this study was implemented to investigate the effects of the biological methyl donor *S*-adenosylmethionine (SAM) on the prostate cancer cell lines PC-3 and Du145. SAM is known to decrease proliferation, migration and invasion of both cell lines [19] but the mechanisms are still not fully clear. However, SAM has proven to be an effective remedy for illnesses like depression or liver diseases without exerting serious side effects, in contrast to chemotherapeutic agents or drugs [3]. Therefore, it seems desirable to gain more information about the mode of action of SAM effects, to explore and establish a new anticancer drug. As antiproliferative effects of SAM are well known, the first steps in this study comprised the examination of cell cycle progression of PC-3 and DU145 cells treated with 200 µm of SAM. A highly significant cell cycle arrest of treated cells in the S-phase and a significant decrease of cancer cells in the G0/G1 phase could be found in both cell lines. Additionally, there was a significant accumulation of treated DU145 cells in the G2/M phase. The cell cycle machinery represents an attractive target for interventions concerning cancer therapies. Numerous chemicals and physical injuries may induce cell cycle arrest at different phases [21]. Agents, damaging the spindle apparatus or the DNA often provoke cell cycle arrest, senescence or apoptosis at the G1/S or G2/M boundaries and have been studied extensively. On the other hand, relatively little is known about cell cycle arrest in the S-phase, during which cells normally duplicate their DNA content [21]. The prevention of DNA synthesis due to an event leading to serious DNA damage, however, could block cells in the S-phase, thus leading to a reduction of tumor cell proliferation and perhaps subsequent apoptosis. The results published in this study are in contrast to previous findings, claiming a cell cycle arrest during the G0/G1 phase in prostate cancer cells treated with SAM [22]. This may be due to differences in the treatment regimen. In the present experiments, cells were treated every 24 h, considering the relatively low half-life time of SAM, which may otherwise lead to the accumulation of SAM metabolites, causing alterations of SAM actions [3]. Cyclin A represents an important factor for cell cycle progression through the S-phase [23], and significant downregulation of the cyclin A protein was observed in PC-3 and DU145 cells treated with SAM for 72 h and 120 h respectively. This is in line with further results, displaying the above-described accumulation of cells in the S-phase following cyclin A downregulation with no further progression in the cell cycle. Nevertheless, since proper cell cycle progression depends on the activity of cyclin-dependent kinases (CDKs), the expression of p21, a protein which is encoded by the *CDKN1A* gene and is known to be a potent inhibitor of several CDKs was examined in this work [24], and not surprisingly, a significant upregulation of p21 protein after 72 h and 120 h of SAM treatment was observed. Though rather linked to cell cycle arrest in G1 and G2 phases, several authors also found an increase in the expression of p21 in conjunction with a cell cycle arrest in the S-phase [24] corroborating these results. Moreover, this seems to not be controversial, since p21 is known to inhibit CDKs, which are necessary for cell cycle progression through the S-phase [24]. Generally speaking, the upregulation of p21 protein may be dependent or independent on the expression of p53 [24]. Since PC-3 cells are hemizygous for chromosome 17, and their single copy of the *p53* gene displays a deletion at codon 138, they express no p53 protein [25]. Thus, in this case, the upregulation of p21 occurs independently from p53 expression. Nevertheless, DU145 cells express the p53 protein, and because the expression of p53 after SAM treatment was not investigated, it is not possible to exclude that SAM exerts antitumorigenic effects via the p53 pathway in DU145 cells, even if the majority of results resembles the findings in PC-3 cells. In general, there are two distinct pathways for the initiation of apoptosis: the death-receptor-induced extrinsic pathway and the mitochondria-apoptosome-mediated pathway, which both require activated caspases. Caspase-3 represents one of the most significant executioner caspases and is known to cleave important cellular proteins, e.g. poly (ADP-ribose) polymerase (PARP), leading eventually to DNA fragmentation and apoptosis [26], and the measurement of the activity of caspase-3 revealed a significant upregulation in both cell lines. Moreover, alterations of the expression of members of the *Bcl-2* gene family, which are located on the mitochondrial membrane, may increase the permeability of the mitochondrial membrane for cytochrome c. This usually results in a cytochrome c release in the cytosol, activating the intrinsic apoptotic pathway. The *Bcl-2* gene family mainly consists of two members: the B-cell lymphoma 2 (bcl-2) protein, essentially involved in the suppression of apoptosis and the Bcl-2 associated X-protein (bax) which is known to promote apoptosis on the other hand [26]. A significant upregulation of bax protein after SAM treatment for 72 h and 120 h was observed in this study, whereas the expression of the bcl-2 protein displayed a moderate downregulation. This leads to a significant increase in the bax/bcl-2 ratio, which normally induces the initiation of the intrinsic apoptotic pathway.

To check whether apoptosis factually occurs in SAM treated cells, an assessment of apoptosis rate using the Muse™ Annexin V and Dead Cell kit was carried out. A moderate but significant increase of early and late apoptosis in SAM treated cells after 72 h and 120 h was found, accompanied by a decrease in the percentage of living cells. Previous reports underpin these findings concerning increased apoptosis, following SAM treatment of cancer cells, partially displaying even higher apoptosis rates [27, 28]. To gain further insights into SAM-dependent anticancer effects, the investigation of important cellular pathways seemed necessary. The MAPK (ERK1/2) signalling pathway and STAT3 pathway have been shown to play pivotal roles in a variety of cellular processes involved in cancerogenesis, including cell cycle regulation, apoptosis, proliferation and migration [28, 29]. The MAPK pathway is an obvious target for SAM, which is known to inhibit the phosphorylation of Extracellular-signal Regulated Kinases in several cancer cell lines [28], and Western blotting confirmed this effect in SAM treated PC-3 cells. Overall, this signalling pathway is considered an essential attack point for numerous therapeutic remedies, and the results of the present study confirmed that SAM is demonstrably included [29]. STAT3 and P-STAT3 are recognised as oncogenes and are highly expressed in several cancer entities as well as cancer cell lines. The upregulation of these two proteins has been associated with higher malignancy rates, poor outcomes [29] and higher expression of genes involved in proliferation, cell survival, angiogenesis, migration and invasion [28, 29]. In contrast, its inhibition leads to a reduction of proliferation, a higher susceptibility to cytotoxic drugs and even to the induction of apoptosis [29]. A significant decrease of STAT3 protein after 72 h and 120 h of SAM treatment could be found, compared to controls and significant downregulation of phosphorylated STAT3 after 72 h and 120 h respectively. In a recent report, STAT3 mRNA was downregulated in PC-3 cells following SAM treatment, which corroborates these results [22].

As a conclusion, one may say, that the treatment of androgen-independent prostate cancer cells with SAM leads to the downregulation of the ERK1/2 pathway and STAT3 pathways which both are involved in cell survival, proliferation, migration and invasion of cancer cells. The downregulation is accompanied by an induction of cell cycle arrest in the S-phase and apoptosis. These effects clearly show that SAM may represent a powerful tool for prostate cancer treatment and is worth further investigation.

## References

[1] Bremmer F, Jarry H, Unterkircher V, Kaulfuss S, Burfeind P, Radzun HJ, Ströbel P, Thelen P (2018). Testosterone metabolites inhibit proliferation of castration- and therapy-resistant prostate cancer. Oncotarget.

[2] Dierks S, von Hardenberg S, Schmidt T, Bremmer F, Burfeind P, Kaulfuß S (2015). Leupaxin stimulates adhesion and migration of prostate cancer cells through modulation of the phosphorylation status of the actin-binding protein caldesmon. Oncotarget.

[3] Lu SC, Mato JM (2012). *S*-Adenosylmethionine in liver health, injury, and cancer. Physiol Rev.

[4] Kotb M, Mudd SH, Mato JM, Geller AM, Kredich NM, Chou JY, Cantoni GL (1997). Consensus nomenclature for the mammalian methionine adenosyltransferase genes and gene products. Trends Genet.

[5] Pajares MA, Markham GD (2011). Methionine adenosyltransferase (*S*-adenosylmethionine synthetase). Adv Enzymol Relat Areas Mol Biol.

[6] Gao J, Cahill CM, Huang X, Roffman JL, Lamon-Fava S, Fava M, Mischoulon D, Rogers JT (2018). *S*-adenosyl methionine and transmethylation pathways in neuropsychiatric diseases throughout life. Neurotherapeutics.

[7] Struck AW, Thompson ML, Wong LS, Micklefield J (2012). *S*-adenosylmethionine-dependent methyltransferases: highly versatile enzymes in biocatalysis, biosynthesis and other biotechnological applications. ChemBioChem.

[8] Finkelstein JD (1990). Methionine metabolism in mammals. J Nutr Biochem.

[9] Chiang PK (1998). Biological effects of inhibitors of *S*-adenosylhomocysteine hydrolase. Pharmacol Ther.

[10] Lu SC (2009). Regulation of glutathione synthesis. Mol Asp Med.

[11] Perez-Leal O, Merali S (2012). Regulation of polyamine metabolism by translational control. Amino Acids.

[12] Heby O (1995). DNA methylation and polyamines in embryonic development and cancer. Int J Dev Biol.

[13] Frostesjö L, Holm I, Grahn B, Page AW, Bestor TH, Heby O (1997). Interference with DNA methyltransferase activity and genome methylation during F9 teratocarcinoma stem cell differentiation induced by polyamine depletion. J Biol Chem.

[14] Avila MA, García-Trevijano ER, Lu SC, Corrales FJ, Mato JM (2004). Methylthioadenosine. Int J Biochem Cell Biol.

[15] Ansorena E, García-Trevijano ER, Martínez-Chantar ML, Huang ZZ, Chen L, Mato JM, Iraburu M, Lu SC, Avila MA (2002). *S*-adenosylmethionine and methylthioadenosine are antiapoptotic in cultured rat hepatocytes but proapoptotic in human hepatoma cells. Hepatology.

[16] Nishikawa K, Iwamoto Y, Kobayashi Y, Katsuoka F, Kawaguchi S, Tsujita T, Nakamura T, Kato S, Yamamoto M, Takayanagi H, Ishii M (2015). DNA methyltransferase 3a regulates osteoclast differentiation by coupling to an *S*-adenosylmethionine-producing metabolic pathway. Nat Med.

[17] Chik F, Machnes Z, Szyf M (2014). Synergistic anti-breast cancer effect of combined treatment with the methyl donor *S*-adenosylmethionine and the DNA methylation inhibitor 5-aza-2′-deoxycytidine. Carcinogenesis.

[18] Li TW, Zhang Q, Oh P, Xia M, Chen H, Bemanian S, Lastra N, Circ M, Moyer MP, Mato JM, Aw TY, Lu SC (2009). *S*-adenosylmethionine and methylthioadenosine inhibit cellular FLICE inhibitory protein expression and induce apoptosis in colon cancer cells. Mol Pharmacol.

[19] Schmidt T, Leha A, Salinas-Riester G (2016). Treatment of prostate cancer cells with *S*-adenosylmethionine leads to genome-wide alterations in transcription profiles. Gene.

[20] Zsigrai S, Kalmár A, Nagy ZB, Barták BK, Valcz G, Szigeti KA, Galamb O, Dankó T, Sebestyén A, Barna G, Szabó V, Pipek O, Medgyes-Horváth A, Csabai I, Tulassay Z, Igaz P, Takács I, Molnár B (2020). *S*-adenosylmethionine treatment of colorectal cancer cell lines alters DNA methylation, DNA repair and tumor progression-related gene expression. Cells.

[21] Williams GH, Stoeber K (2012). The cell cycle and cancer. J Pathol.

[22] Shukeir N, Stefanska B, Parashar S, Chik F, Arakelian A, Szyf M, Rabbani SA (2015). Pharmacological methyl group donors block skeletal metastasis in vitro and in vivo. Br J Pharmacol.

[23] Yam CH, Fung TK, Poon RYC (2002). Cyclin A in cell cycle control and cancer. Cell Mol Life Sci.

[24] Karimian A, Ahmadi Y, Yousefi H (2016). Multiple functions of p21 in cell cycle, apoptosis and transcriptional regulation after DNA damage. DNA Repair (Amst).

[25] Chappell WH, Lehmann BD, Terrian DM, Abrams SL, Steelman LS, McCubrey JA (2012). p53 expression controls prostate cancer sensitivity to chemotherapy and the MDM2 inhibitor Nutlin-3. Cell Cycle.

[26] Li Y, Yu H, Wang M, Luo Y, Guo X (2018). Biochanin A induces S phase arrest and apoptosis in lung cancer cells. Biomed Res Int.

[27] Mahmood N, Cheishvili D, Arakelian A, Tanvir I, Khan HA, Pépin AS, Szyf M, Rabbani SA (2018). Methyl donor *S*-adenosylmethionine (SAM) supplementation attenuates breast cancer growth, invasion, and metastasis in vivo; therapeutic and chemopreventive applications. Oncotarget.

[28] Ilisso CP, Sapio L, Delle Cave D, Illiano M, Spina A, Cacciapuoti G, Naviglio S, Porcelli M (2016). *S*-adenosylmethionine affects ERK1/2 and Stat3 pathways and induces apotosis in osteosarcoma cells. J Cell Physiol.

[29] Zhu Q, Hu J, Meng H, Shen Y, Zhou J, Zhu Z (2014). S-phase cell cycle arrest, apoptosis, and molecular mechanisms of aplasia ras homolog member I-induced human ovarian cancer SKOV3 cell lines. Int J Gynecol Cancer.

